# Effect of Inquiry-Based Stress Reduction (IBSR) Intervention on Well-Being, Resilience and Burnout of Teachers during the COVID-19 Pandemic

**DOI:** 10.3390/ijerph18073689

**Published:** 2021-04-01

**Authors:** Tzofnat Zadok-Gurman, Ronit Jakobovich, Eti Dvash, Keren Zafrani, Benjamin Rolnik, Ariel B. Ganz, Shahar Lev-Ari

**Affiliations:** 1 Department of Health Promotion, Sackler Faculty of Medicine, School of Public Health, Tel-Aviv University, Tel-Aviv 69978, Israel; tzofnatzadok@mail.tau.ac.il (T.Z.-G.); drronit@tauex.tau.ac.il (R.J.); 2 The Israeli Ministry of Education, Jerusalem district, Jerusalem 95464, Israel; eti.dvash@gmail.com; 3 Begin High School, John Kennedy Street 8, Rosh Ha’Ayin 4852028, Israel; kerenzafrani25@gmail.com; 4 Department of Genetics, Stanford University, Stanford, CA 94305, USA; rolnik@stanford.edu (B.R.); abganz@stanford.edu (A.B.G.)

**Keywords:** IBSR, teachers, well-being, COVID-19, personal health promotion

## Abstract

**Objective:** The COVID-19 pandemic has had a major impact on teachers professional and personal lives. Our primary aim was to assess the effect of a blended Inquiry-Based Stress Reduction (IBSR), an emerging mindfulness and cognitive reframing intervention on teacher’s well-being. Our secondary aims were to assess the effect of IBSR on resilience, burnout, mindfulness, and stress among teachers during the COVID-19 pandemic. **Methods:** The study was a prospective controlled trial with an intervention group (N = 35) and a comparison control group (N = 32). The intervention took place in the Jerusalem District throughout the school year from November 2019 to May 2020. The sessions were conducted in blended learning that included traditional learning (face-to-face) and online learning. Data was analyzed on an intention-to-treat basis. **Results:** IBSR blended intervention enhanced the resilience and improved the subjective and psychological well-being of teachers in spite of the breakout of the COVID-19 pandemic and the first lockdown in Israel. Simultaneously the control group suffered from enhanced burnout levels and a decline in psychological and subjective well-being. **Conclusions:** Implementation of IBSR blended intervention during the school year may benefit teachers’ well-being and ability to flourish, even during stressful events such as the COVID-19 pandemic.

## 1. Introduction

The Coronavirus Disease 2019 (COVID-19) pandemic caught the Israeli education system by surprise. When Israel implemented social distancing restrictions, teachers had to rapidly adapt to a new format of distance learning. The pandemic has exposed teachers to many new challenges, such as increasing workloads, the need to learn and utilize new technologies, changes to daily teaching routines, fear of COVID-19, and uncertainty around how long distance learning will last, all while coping with full-time work from home [1,2,3,4,5]. Furthermore, the changes at work were accompanied by physical distancing from friends, colleagues, and students. The psychological and subjective effects on teachers’ well-being as a result of quarantine and social distancing may manifest as psychological distress, anxiety and depression [4,5,6,7]. Such negative outcomes have been shown to be reduced in teachers who participated in psycho-social and mindfulness-based interventions [6,8,9]. However, such interventions are typically performed in a face-to-face context. With the limits imposed by the COVID-19 pandemic on face-to-face interventions, the impact of online remote and blended interventions, such as IBSR, on teacher well-being during the pandemic lockdown are of particular interest.

Job burnout has been researched intensively in the field of education, and various studies have described its prevalence among teachers [10,11,12]. Burnout is manifested by a loss of energy, resources, and physical and mental forces, and usually, it is a result of prolonged stress at work or emotional overload, and high demands [13,14]. In the 1980s, Maslach defined a model of burnout comprising three components: emotional exhaustion, reduced personal accomplishment, and depersonalization [15,16,17]. Burnout is a chronic and progressive state [16,18,19]. Recently, in May 2019, the World Health Organization (WHO) set out to include burnout as an occupational phenomenon in the 11th Revision of the International Classification of Diseases (ICD-11) [20].

In recent years, there is growing evidence regarding the effectiveness of specific interventions for burnout and mental problems in teachers, such as Cognitive Behavioral Therapy (CBT), Positive Psychology Interventions (PPI) and mindfulness, transcendental meditation, prayer, breathing and yoga [21,22,23,24]. A cohort study was conducted on 66 Italian teachers who underwent an intervention that included an 8-week mindfulness-based stress reduction (MBSR) course. The course consisted of offline and online sessions. The teachers were asked a month before and a month after the lockdown in Italy about their attention skills, empathy, personality profiling, awareness of perception, psychological well-being, emotional distress, and levels of burnout. The study found that MBSR intervention can effectively reduce the negative psychological implications of the COVID-19 pandemic, helping to improve teacher resilience and well-being during the lockdown [6]. A recent study assessed the efficacy of information and communications (ICT) technology training program on teacher’s stress and burnout levels during COVID-19 pandemic. The study that was conducted on primary school teachers is Spain found that participants in the training showed a decreased stress and burnout levels and an increase in the emotional intelligence levels [25]. While these studies showed promising results, in specific contexts, there is a scarcity of research on the effectiveness of blended interventions that combine offline and online sessions for improving teachers’ well-being and resilience during critical events and crises, such as the COVID-19 pandemic.

Inquiry Based Stress Reduction (IBSR) is an interventional adaptation of “The Work,” a self-inquiry method (thework.com) developed in the U.S. in 1986 by Byron Katie. This method has been practiced by hundreds of thousands of people from 30 countries around the world [26]. IBSR is based on principles and skills of observation, self-exploration, change of interpretation (cognitive reframing), and empowerment. This technique teaches participants to systematically identify the stressful thoughts that provoke anxiety. The participant explores the thoughts meditatively to try to experience a different interpretation of the reality he experiences. The process may result in increasing a person’s internal motivation for change, raising self-capacity, and reenforcing thought patterns that empower participants to adopt health behaviors of a healthy lifestyle [26,27,28].

IBSR shares the same fundamental assumption as classical cognitive behavioral therapy (CBT), namely that dysfunctional beliefs are the main cause of distress. However, the process of cognitive restructuring involved in IBSR is addressed by awareness and personal realization rather than reasoning and argument as in CBT [29,30,31]. Also, unlike CBT, IBSR can be practiced alone or with others and does not require a trained facilitator. This technique does not require any intellectual, religious, or spiritual preparation, but rather a desire to deepen self-awareness [26].

IBSR has been tested on a range of populations around the world. It was found to elevate mental health and wellbeing among cancer patients and BRCA1/2 carriers [32,33]. IBSR was also tested in a nonclinical population, and it was shown to be effective in improving depression and anxiety, and enhancing happiness and wellness [34,35]. A previous controlled trial that investigated the effect of IBSR on teacher burnout found that IBSR may be a viable approach to reducing teacher burnout [8,9]. The qualitative aspect of the study showed that participants in the intervention group experienced a greater ability to accept reality [9]. The quantitative part of the study showed that burnout parameters of emotional exhaustion and personal accomplishment improved significantly in the intervention group, as compared with the control group [8].

The main aim of our study was to assess the effects of an IBSR blended intervention on psychological and subjective well-being. Our secondary aims were to assess the effects of IBSR blended intervention on resilience, burnout, mindfulness, and teachers’ stress during the COVID-19 pandemic lockdown period.

## 2. Methods

### 2.1. Recruitment and Study Procedure

A non-randomized controlled trial with intervention and comparison groups was carried out in two large cities in Israel from the Jerusalem district in cooperation with the Ministry of Education (MOE). Recruitment for the intervention group (N = 32) was done through the sites of the PISGA Jerusalem center and Modi’in, with online enrollment. The control group (N = 28) included teachers that participated in other courses in the same centers, which are held in parallel to the intervention group’s course, and also teachers from a school in Jerusalem. The teachers were eligible to participate in the study if they agreed to sign an informed consent form and had no previous experience with the IBSR technique. The study was approved by both the ethics Committee of Tel-Aviv University (Approval- 0000554-1) and the Israeli Ministry of Education (Approval- 10985). To reduce dropout rates from the control group, participants who continued through the end were given an IBSR book at completion of the study.

### 2.2. Intervention

**Figure 1:**
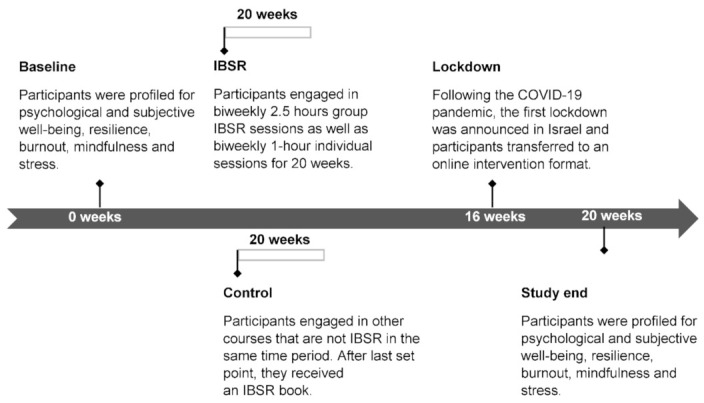
Study Timeline. Participants were profiled at baseline for psychological and subjective well-being, resilience, burnout, mindfulness and stress. They were engaged in IBSR or control and underwent either a 20-weeks IBSR training program or a 20-weeks waiting period. Psychological profiling and assessment of burnout were performed again at study week 20.

The IBSR intervention included 10 biweekly group meetings (2.5 h/meeting) and biweekly individual sessions with a facilitator (1 h/session) for 20 weeks. All of the sessions were standardized according to a training manual and assessed to maintain consistency in the program. As of March 2020, the intervention program was moved to an online format following the first lockdown in Israel due to the COVID-19 pandemic. The intervention method was a blend that combined offline learning (face-to-face) and online learning sessions (Figure 1). Participants were considered active if they were present in at least 80% of the group meetings and completed 50% of the home practice.

IBSR technique involves three steps: In the first, step participants identify stressful thoughts and write these stressful thoughts out on paper. The main tool for this task is the “*Judge-Your-Neighbor*” worksheet (Supplementary Material Table S1). In the second step, participants “inquire” into their stressful thoughts. During the process of inquiry, the participants, on their own or with the help of a facilitator (a person trained in the IBSR technique), investigate their stressful thoughts using a set of four guided questions: (a) Is it true? (b) Can I absolutely know that it is true? (c) How do I react when I believe that thought? (d) Who would I be without the thought? The self-investigation enables the participant to question their automatic thoughts and examine their emotional and physical responses during stress-evoking situations. This stage is meditative, and the participants are encouraged to identify their own true answers to the four questions with no pre-defined agenda. The guidance is to be in a state of witnessing awareness, in which a person observes the thoughts that come into the mind without trying to control or direct them. The goal is realization, not rationalization. In the final step, participants “turn around” their stressful thoughts. In the turnarounds, participants identify possible evidence for the opposite of the thought [26]. For example, if the original thought was: “My students don’t listen to me,” possible turnarounds can be: “I don’t listen to my students” (turnaround to the other), “I don’t listen to myself” (turnaround to the self), and “My students do listen to me” (turnaround to the opposite).

### 2.3. Data Collection

All participants completed the following questionnaires before (study baseline) and after the intervention (study completion at week 20). Surveys were given and collected online included the following validated questionnaires:

- The PERMA Profiler—in the book Flourish (2011), Seligman defined well-being in terms of five pillars: Positive emotion, Engagement, Relationships, Meaning, and Accomplishment, or PERMA [36]. Butler & Kern [37] developed the PERMA-Profiler as a brief measure of PERMA. The questionnaire includes 23 items: 3 questions from every 5 domains, and additionally, 3 questions around negative emotions and health and 1 question about loneliness and overall well-being.
- Satisfaction with Life Scale (SWLS)—Since its introduction in 1985, the Satisfaction with Life Scale by Diener, Emmons, Larsen & Griffin [38] has been regularly used as a measure of the life satisfaction component of subjective well-being [39]. The SWLS consists of 5-items that require a rating on a 7-point Likert scale [40].
- Positive and Negative Affect Scale (PANAS)—evaluates the emotional state of individuals and includes 10 items of positive affect and 10 items of negative affect. The questionnaire was used as a measure of the emotional component of subjective well-being, and was found consistent on various time points; hence, it can be used as a state or trait scale [41]
- The Brief Resilience Scale (BRS)—The brief resilience scale was created to assess the ability to bounce back or recover from stress [42]. The questionnaire includes 6 questions: 3 positively worded and 3 negatively worded, using a Likert scale between 1–5.
- Mindfulness in Teaching Scale (MITS)—The questionnaire includes 20 items, a mixture of new unique items and items modified from existing scales (e.g., MAAS). Included items are teacher focus during instruction daily school activities, emotional awareness, self-regulation, and responsivity and sensitivity during student-teacher interactions. The instructors asked teachers to read statements that describe how true each statement was for them within the past month on a 1 to 5 Likert scale [43].
- Maslach Burnout Inventory (MBI)—the MBI is the most commonly used questionnaire in the field of occupational burnout in the last 30 years [44,45]. Its validity and reliability were demonstrated in various studies [16,19]. The MBI includes 14 items, which evaluate emotional exhaustion (8 items) and personal accomplishment (6 items).
- Perceived Stress Scale (PSS)—The PSS evaluates the frequency in which a person perceives daily situations as stressful. It includes 14 items rated on a scale of 0 to 4. A higher score signifies a more stressful daily experience [46].
- Demographic questionnaire—includes demographic questions such as age, economic status, marital status, years of education, years of teaching, teaching role at the school, and the degree of spirituality.

### 2.4. Data Analysis

An intention-to-treat analysis approach was implemented for this study. Prior to study start, a sample size calculation was done using WinPepi software (version 11.65) on emotional exhaustion variables with an expected difference of 3.5 units between groups and a standard deviation of 4.5 units for each group [8]. A required sample size of 52 participants was calculated in order to achieve an alpha of 5% and 80% power. The similarity of baseline (T1) demographic characteristics of participants in the control and intervention groups was assessed by independent *t*-tests for continuous variables and chi-square tests for categorical variables between groups. A sensitivity analysis with gender was conducted to assess differences in baseline outcomes between male and female participants. Pearson correlation coefficients were used to examine the correlation between the different instruments at baseline. A two-way mixed-effects model (with both fixed and random effects) for repeated measures was used to examine interactions between group and time factors. The dependent variable was the psychosocial variable and independent (fixed) variables were time (before and after intervention), group, and the interaction time by group. Sensitivity analysis of mixed models with outcomes measures at baseline (T1) as covariates was conducted to control for the differences in variables between the groups. To assess differences between groups dropout bias, a comparison was carried out between participants who completed the intervention and those who dropped out. To test the missing complete and random (MCAR) assumption, we applied Little’s test [47], which divides the sample into groups based on the patterns of missingness for the study outcome. The statistical software SPSS 26 (IBM, New York City, NY, USA) was used for all the data processing within this study.

## 3. Results

### 3.1. Study Cohort and Baseline Demographics Characteristics

Sixty-seven teachers agreed to participate in the study. Table 1 details the demographic characteristics of the control (n = 32) and intervention group (n = 35). Most of the study population (86.6%) were females. The mean age of the participants was 45 years. All the subjects had completed 12 years of education, and 74.6% were married. According to the collected demographic data, there were no significant differences between the control and the intervention groups. Seven teachers (3 from the intervention and 4 from the control group) only completed the initial questionnaire. To evaluate whether data from the dropouts were missing at random or due to a systemic error, we implemented the Little’s MCAR test (X^2^ = 146.1, *p* = 0.157). No significant differences in baseline metrics were identified between dropouts and completers (Supplementary Material Table S2) and therefore, they were not included in further analysis.

### 3.2. Teachers’ Psychological and Subjective Well-Being Pre-Post IBSR Intervention

**Figure 2:**
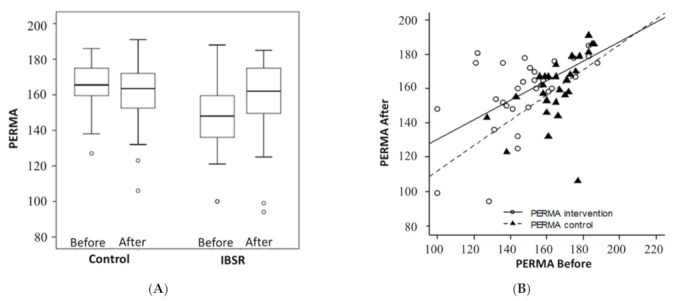
(**A**)**.** Effect of IBSR intervention on the psychological well-being (PERMA) in comparison to control group. Data is displayed as box plot of both groups. * *p* < 0.05. (**B**)**.** Relationship between the pre- and post-PERMA profiler for the two groups. Scatter plot analysis indicate that IBSR (solid line) was more effective than control (dashed line), especially for subjects with low initial PERMA scores.

Table 2 describes the effects of the IBSR intervention among teachers during the COVID-19 pandemic. Mixed model analysis was adjusted for the outcome measurement made at T1, and effect size was calculated for each variable. According to the data collected within our study, there was a marked improvement in the teacher’s psychological well-being (PERMA) (Figure 2), in the intervention group (147.8 ± 21.6 to 158.1 ± 21.7), and a slight decrease in the control group (165.6 ± 13.8 to 160.5 ± 19.3). The difference between the groups was statistically significant (*p* < 0.01) with a large effect size (Cohen’s d = 0.806).).

**Figure 3:**
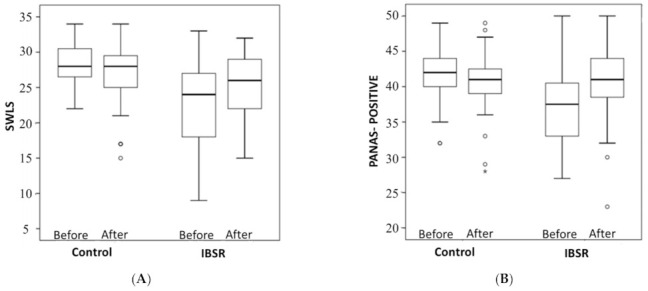

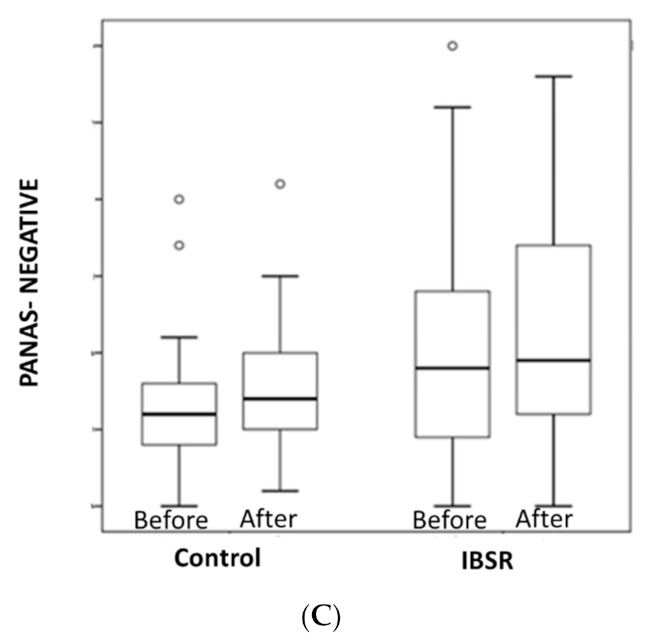
(**A**) Effect of IBSR intervention on Satisfaction with Life Scale (SWLS) in comparison to control group. Data is displayed as box plot of both groups. * *p* < 0.05. (**B**). Effect of IBSR intervention on Positive Affect sub-scale (PANAS-positive) in comparison to control group. Data is displayed as box plot of both groups. * *p* < 0.05. (**C**). Effect of IBSR intervention on Negative affect sub-scale (PANAS-negative) in comparison to control group. Data is displayed as box plot of both groups. * *p* < 0.05.

The teachers’ subjective well-being, measured by the satisfaction with life scale (SWLS) (Figure 3A), was significantly improved in the intervention group (22.9 ± 5.8 to 25.2 ± 4.7), as compared to the control group where it declined (28.3 ± 3.1 to 26.2 ± 4.9). The difference between the groups was statistically significant (*p* < 0.01). In the emotional component (PANAS) of subjective well-being (Figure 3B), an increase was found in the positive emotions of the intervention group (37.4 ± 5.5 to 40.2 ± 5.7), while a slight decrease was found in the control group (41.6 ± 4.3 to 40.4 ± 4.8). This difference between groups was statistically significant (*p* < 0.01). A large difference between the groups was found in satisfaction with life (Cohen’s d = 0. 0.934) and positive emotion (Cohen’s d = 0.908) outcomes. The change in negative affect (Figure 3C) between the groups was not significant.

### 3.3. Teachers’ Resilience, Burnout, Mindfulness, and Stress Pre-Post IBSR Intervention

Teachers in the intervention group indicated positive improvement in resilience score (BRS) (3.0 ± 0.9 to 3.2 ± 0.8), while a decrease in resilience levels was noted in the control group (3.7 ± 0.6 to 3.5 ± 0.5). The difference between the groups was statistically significant (*p* = 0.04). The level of mindfulness increased in the intervention group (72.0 ± 6.3 to 75.1 ± 7.3), while no change was seen in the control group (77.3 ± 7.2 to 77.2 ± 7.9). The difference between the groups was statistically significant (*p* = 0.049).

Teachers in both intervention (12.7 ± 5.9 to 18.3± 5.7) and control (9.7 ± 4.9 to 18.6± 4.5) groups showed deterioration in emotional exhaustion. However, the increase in the intervention group was less substantial than in the control group. The difference between the groups was statistically significant (*p* < 0.01). A large difference between the groups was found in emotional exhaustion (Cohen’s d = 0.752), while medium differences were found in resilience (Cohen’s d = 0.549) and mindfulness (Cohen’s d = 0.524) outcomes. In the personal accomplishment scales, no change was found in the intervention group (10.8 ± 5.9 to 10.7 ± 4.9), compared to an increase in the control group (7.3 ± 3.9 to 9.3 ± 3.6). This difference was borderline significant (*p* = 0.051). In the daily perceived stress scales, we did not observe differences between the groups.

The participants age in our study ranged between 34 to 67 (median age = 46, mean age = 45). Since research shows that burnout levels may differ between younger and elder teachers, we performed a sensitivity analysis based on our median age in the outcome variables. Our analysis did not find differences in baseline outcomes between younger or elder teachers (Supplementary Material Table S3), and the effect of the intervention was similar between groups.

### 3.4. Correlations Analysis and Sensitive Analysis of the Study Measures

Table 3 shows positive correlations between psychological well-being (PERMA) and subjective well-being (SWLS; r = 0.443, *p* < 0.01), positive emotions (PANAS-*p*; r = 0.662, *p* < 0.01), mindfulness (MITS; r = 0.431, *p* < 0.01), and resilience (BRS; r = 0.316, *p* = 0.02).

Negative correlations were found between psychological well-being and negative emotions (r = −0.298, *p* = 0.02).

Since different confrontation strategies to stress or anxiety may differ between males and females, we performed a sensitive analysis to adjust for possible differences between genders in the outcome variables. Our analysis did not find differences in baseline outcomes between genders (Supplementary Material Table S4).

### 3.5. Internal Consistency of the Trial Instruments

Cronbach’s Alpha coefficients were calculated independently for each of the instruments (Supplementary Material Table S5). High internal consistency was revealed (α ranged between 0.707 to 0.877), indicating a relatively high degree of probability that the items within each instrument were addressing the same constructs.

## 4. Discussion

The COVID-19 pandemic has posed unique challenges for teachers. In the present study, school teachers who participated in an IBSR blended intervention, in spite of the breakout of the COVID-19 pandemic and the first lockdown in Israel exhibited increases in psychological and subjective well-being, mindfulness, and resilience. In contrast, the control group suffered from a decline in resilience and psychological and subjective well-being, and enhanced levels of emotional exhaustion. Positive correlations were found between psychological well-being, subjective well-being, resilience, and mindfulness (r = 0.39–0.66 *p* < 0.01).

Subjective well-being (SWB) consists of three primary components: life satisfaction, positive affect, and negative affect. Positive affect consists of pleasant emotions and feelings such as joy and happiness, whereas negative affect consists of unpleasant emotions or feelings such as sadness and fear. Life satisfaction refers to a cognitive, judgmental process- a global assessment of one’s life as a whole. SWB allows people to judge their lives based on their values and standards [38,48]. Several studies have shown the COVID-19 has had a major effect on SWB. A cohort study that examined changes in subjective well-being of 887 German participants between December 2019 and May 2020 at four time points found a decrease in life satisfaction and positive affect and an increase in negative affect among participants between the beginning of the lockdown and at the end (March 2020 and May 2020) [49]. The present study assessed the effect of an IBSR intervention on the three components of the SWB during the COVID-19 pandemic, as compared to the control group. Teachers’ life satisfaction increased in the intervention group despite the pandemic and the enforced social distancing measures (22.9 ± 5.8 to 25.2 ± 4.7, *p* = 0.047), while the control group experienced a decline in life satisfaction during this period (28.3 ± 3.1 to 26.2 ± 4.9, *p* < 0.01). The difference between the groups was significant (*p*< 0.01). These findings are in accordance with a previous study that found a positive effect of IBSR on happiness levels in a general population of 197 adults, which increased significantly after the IBSR intervention [35].

Positive affect (37.4 ± 5.5 to 40.2 ± 5.7, *p* < 0.01) was enhanced in the IBSR intervention group while it declined in the control group (41.6 ± 4.3 to 40.4 ± 4.8, *p* = 0.049). The difference between the groups was significant (*p*< 0.01). The COVID-19 pandemic challenged people to adapt to rapid changes in their daily habits and face high levels of uncertainty. A cross-sectional study of 541 Spanish adults, which examined reactions to the COVID-19 outbreak and its impact on SWB, found that high levels of positive affect and low levels of negative affect might help individuals to adopt information-processing strategies during the COVID-19 outbreak that will improve their life satisfaction. Results showed a positive correlation between positive affect and life satisfaction (r= 0.21, *p* < 0.005) [50]. However, changes in the participants’ negative affect were not detected in either group. It could have been caused by the fact that, despite the increase in the satisfaction and positive emotions in the intervention group, the COVID-19 pandemic and prolonged social distancing had a dramatic negative impact across many domains of the teachers’ personal lives [51,52].

Psychological well-being is characterized by a sense of fulfillment, happiness, and meaning. Seligman et al., developed a validated model of psychological well-being constructed of five pillars: Positive emotion, Engagement, Relationships, Meaning, and Accomplishment (PERMA) [36,37]. Positive correlations were found between PERMA and resilience to stressful events [53]. Dimitra et al. found that the imposed stay-at-home and social distancing regulation during the COVID-19 pandemic decreases psychological well-being measured by the PERMA profiler [54]. Our study assessed the effect of IBSR intervention on psychological well-being (PERMA) during the COVID-19 pandemic, compared to the control group. Teachers’ psychological well-being and happiness increased in the intervention group despite social distancing and a general lockdown (147.8 ± 21.6 to 158.1 ± 21.7, *p* = 0.01), while the control group experienced a decline in PERMA during this period (165.6 ± 13.8 to 160.5 ± 19.3, *p* = 0.12). The difference between the groups was significant (*p*< 0.01). These findings are in concurrence with a pilot study that found a positive effect of Positive Psychological Intervention (PPI) on 20 health care professionals’ workplace-well-being and flourishing. At post-course, each of the psychological well-being domains scores increased. The increase in participants’ overall PERMA-H Workplace Well-being score (6.87 ± 1.56 to 7.29 ± 1.33, *p* = 0.05) was primarily due to increases in Positive Emotion (*p*= 0.02) and Meaning (*p*= 0.03) domains, of which each was significant [55].

Resilience is defined as the process of adapting well in the face of stress, adversity, trauma, tragedy, and threats [56]. Other definitions include the “ability to bounce back” quickly from stressful situations and flexible adaptation to a new situation [57]. Resilience can be considered a personal strength that can contribute to positive functioning and optimal development, and prevent negative emotions, thoughts, and behaviors [58]. Resilience can reduce the adverse effects of stress factors on mental health and promote positive mental health in difficult times, such as a pandemic. Yildirim et al. investigated the mediating effect of resilience on the relationship between stress and burnout among 402 Turkish adults during the COVID-19 pandemic. Burnout was positively related with stress (r = 0.71, *p* < 0.001), while resilience was negatively related to burnout (r = −0.56, *p* < 0.001) and stress (r = −0.54, *p* < 0.001) [58]. Our results showed positive correlations between resilience and psychological well-being (r = 0.32, *p* = 0.01), life satisfaction (r = 0.39, *p* < 0.01), and positive affect (r = 0.31, *p* = 0.02) and negative correlations with emotional exhaustion (r = −0.316, *p* < 0.01).

After the Intervention, the control group exhibited a decrease in resilience (3.7 ± 0.6 to 3.5 ± 0.5, *p* = 0.06) while the IBSR group demonstrated increased resilience (3.0 ± 0.9 to 3.2 ± 0.8, *p* = 0.19), and the difference was significant between the groups (*p* = 0.04). The IBSR participants improved their abilities to deal with stressful situations during the COVID-19 pandemic. A cohort study investigated whether MBSR can lead to an increase in resilience in 49 participants, MBSR intervention group and control group. It found that MBSR participants showed an increase in post-intervention resilience (2.76 ± 0.60 to 3.13 ± 0.72), in contrast to the control group (3.09 ± 0.82 to 3.06 ± 0.70). The difference was significant between the groups (*p* < 0.01) [59].

Mindfulness is one of the key components of IBSR. During the practice of IBSR, participants learn to be mindful of the automatic thoughts that cause suffering, to observe their attachment to these thoughts, and to internally inquire whether these thoughts are indeed true [26]. Our results indicate that mindfulness increased among the IBSR group (72.0 ± 6.3 to 75.1 ± 7.3), while it remained unchanged in the control group (77.3 ± 7.2 to 77.2 ± 7.9). The difference between the groups was significant (*p*= 0.047). Differences in perceived stress and personal accomplishment between the groups over time were not observed. Other studies justify the finding that personal accomplishment increases during the COVID-19 pandemic [60,61]. For example, a Canadian study assessed the burnout levels of 1626 teachers during the COVID-19 pandemic. It found that over the first three months of the pandemic, teachers demonstrated an increased sense of personal accomplishment [61]. Emotional exhaustion increased in both groups, but the increase was significantly higher in the control group compared to the intervention (*p* < 0.01).

Several studies have shown that teachers at elder age and more years of service are prone to elevated levels of emotional exhaustion, and lower levels of well-being [62,63]. In our study, the participants median age was 46 with average of 17 years in the teaching profession. Our findings demonstrate that IBSR blended intervention enhanced the resilience and improved the subjective and psychological well-being of participants, while the control group suffered from a decline in psychological and subjective well-being and enhanced levels of burnout. Our sensitive analysis did not find differences in baseline outcomes between younger or elder teachers (Supplementary Material Table S3), and the effect of the intervention was similar between groups. More research with a larger sample, and with different age groups of teachers are required to confirm these finding and contribute to evidence-based practice.

We are aware of several limitations in the current study. First, the assignment was not randomized; however, it should be noted that a comparison between the intervention and the control group did not reveal any difference between participant’s characteristics on baseline; and a sensitivity analysis of mixed models with outcomes measures at baseline (T1) as covariates was conducted to control for possible differences in variables between the groups. Second, most of the participants in the study were female (86.6%) which are reported to experience higher work-related anxiety, more fear of COVID-19, and depression than male teachers [1,4,5,6]. However, we did not find differences between male and female participants in study outcomes. Studies with a larger sample of male teachers are required to generalize these findings to both genders. Third, study outcomes were based on subjective outcomes that may be affected by response bias. Although using validated questionnaires and keeping the anonymity of participants may minimize this effect, future studies should assess the randomized controlled design and effect of IBSR also on objective outcomes related to health behaviors and health status of participants. Finally, the study did not include a longitudinal follow-up period; A follow-up assessment is needed to evaluate whether the improvements of the study outcomes remain in the long term, and during various stages of the pandemic. This assessment is important because IBSR can be practiced alone or with others and does not require a trained facilitator. Whether participants continue to practice the IBSR on their own is a meaningful factor in the successful implementation of this intervention.

## 5. Conclusions

We have shown that an IBSR blended intervention enhanced the resilience and improved the subjective and psychological well-being of teachers in spite of the breakout of the COVID-19 pandemic and the first lockdown in Israel. Simultaneously the control group suffered from enhanced burnout levels and a decline in psychological and subjective well-being. The results of the current study demonstrate that an IBSR intervention may be a viable approach to enhancing teachers’ well-being and resilience, as well as the ability to flourish, even during stressful events such as the COVID-19 pandemic. Future randomized controlled studies are warranted to assess the potential efficacy of the technique as a blended tool for improving well-being among teachers and other stressed-out workers in general, or during crises such as the COVID-19 pandemic.

## Declaration of Interest

None.

## Figures and Tables

**Table 1:** Baseline Demographic (N = 67).

		Intervention Group (N = 35)	Control	Difference between Groups	
			Group (N = 32)		
		Mean (SD)	Mean (SD)	Test Statistic	*p*-Value
**Age (yrs)**		46.9 (8.9)	42.9 (9.2)	T = 2.79	0.078
**Education (yrs)**		17.1 (3.2)	18.2 (3.5)	T = 1.35	0.182
**Seniority ^b^ (yrs)**		17.7 (9.7)	14.6 (9.8)	T = 1.30	0.198
**Job percent (%)**		91.3 (13.2)	93.7 (14.3)	T = 0.69	0.49
**Gender ^a^**	Female	33 (94.3%)	25 (78.1%)	X^2^ = 3.75 ^a^	0.053
	Male	2 (5.7%)	7 (21.9%)		
**Marital status ^a^**	Single	4 (11.4%)	3 (9.4%)	X^2^ = 2.37 ^a^	0.668
	Married without children	2 (5.7%)	1 (3.1%)		
	Married with children	23 (65.7%)	24 (75.0%)		
	Divorced	6 (17.1%)	3 (9.4%)		
	widowed	0 (0.0%)	1 (3.1%)		
**Economic ^a^**	Below average	2 (5.7%)	4 (12.5%)	X^2^ = 0.97 ^a^	0.616
	Average	23 (65.7%)	19 (59.4%)		
	Above average	10 (28.6%)	9 (28.1%)		

Abbreviations: SD, standard deviation; df, degrees of freedom. ^a^ N (% within group). ^b^ How many years have you been in the teaching profession?

**Table 2:** Effects of IBSR intervention between the study groups.

		Intervention Group (N = 32)	Control Group (N = 28)	Mixed Effect Model (Time × Group)		Effect Size
		Mean (SD)	Mean (SD)	Test Statistic (Time × Group)	*p*-Value	Cohen’s d
**PERMA ^b^**	Before	147.8 (21.6)	165.6 (13.8)	F = 9.55 (Increase)	0.003 **	0.806
	After	158.1 (21.7)	160.5 (19.3)			
**SWLS ^c^**	Before	22.9 (5.8)	28.3 (3.1)	F = 12.64 (Increase)	0.001 **	0.934
	After	25.2 (4.7)	26.2 (4.9)			
**PANAS-P ^a^**	Before	37.4 (5.5)	41.6 (4.3)	F = 12.09 (Increase)	0.001 **	0.908
	After	40.2 (5.7)	40.4 (4.8)			
**PANAS-N ^a^**	Before	20.1 (6.7)	16.7 (4.5)	F = 0.08 (Increase)	0.783	0.072
	After	21.5 (7.7)	17.3 (4.9)			
**BRS ^b^**	Before	3.0 (0.9)	3.7 (0.6)	F = 4.44 (Increase)	0.039 *	0.549
	After	3.2 (0.8)	3.5 (0.5)			
**MBI EE**	Before	12.7 (5.9)	9.7 (4.9)	F = 8.45 (decrease)	0.005 **	0.752
	After	18.3 (5.4)	18.6 (4.5)			
**MBI PA**	Before	10.8 (5.9)	7.3 (3.9)	F = 3.97 (decrease)	0.051	0.516
	After	10.7 (4.9)	9.3 (3.6)			
**MITS**	Before	72.0 (6.3)	77.3 (7.2)	F = 4.10 (Increase)	0.047 *	0.524
	After	75.1 (7.3)	77.2 (7.9)			
**PSS**	Before	33.9 (10.8)	39.5 (8.8)	F = 1.25 (Increase)	0.269	0.289
	After	36.0 (9.4)	39.0 (8.0)			

Abbreviations: PERMA, positive emotion, engagement, relationships, meaning, and accomplishment; SWLS, Satisfaction with Life Scale; PANAS, Positive and Negative Affect Scale; BRS, Brief Resilience Scale; MBI, Maslach Burnout Inventory, EE—emotional exhaustion, PA—personal accomplishment; MITS, Mindfulness in Teaching Scale; PSS, Perceived Stress Scale; SD, standard deviation. * *p*-value of <0.05 indicating statistical significance. ** *p*-value of < 0.01 indicating statistical significance ^a^ N (control) = 27. ^b^ N (intervention) =31. ^c^ N (intervention) = 30.

**Table 3:** Pearson correlation between measures at baseline (n = 60).

	MBI EE	MBI PA	MITS	PANAS-*p*	PANAS-N	PSS	PERMA	BRS	SWLS
**MBI EE**	1								
**MBI PA**	0.657 ** <0.001	1							
**MITS**	−0.314 * 0.015	−0.294 * 0.023	1						
**PANAS-P**	−0.571 ** <0.001	−0.451 ** <0.001	0.398 ** 0.002	1					
**PANAS-N**	0.463 ** <0.001	0.116 0.381	−0.177 0.181	−0.377 ** 0.003	1				
**PSS**	0.576 ** <0.001	0.341 ** 0.008	−0.303 * 0.019	−0.549 ** <0.001	0.431 ** 0.001	1			
**PERMA**	−0.523 ** <0.001	−0.546 ** <0.001	0.431 ** 0.001	0.662 ** <0.001	−0.298 * 0.023	−0.487 ** <0.001	1		
**BRS**	−0.316 * 0.015	−0.042 0.753	0.315 * 0.015	0.308 * 0.019	−0.237 0.073	−0.492 ** <0.001	0.316 * 0.015	1	
**SWLS**	−0.347 ** 0.008	−0.461 ** <0.001	0.287 * 0.029	0.366 ** 0.005	−0.092 0.497	−0.592 ** <0.001	0.443 ** <0.001	0.389 ** 0.003	1

Abbreviations: PERMA, positive emotion, engagement, relationships, meaning, and accomplishment; SWLS, Satisfaction with Life Scale; PANAS, Positive and Negative Affect Scale; BRS, Brief Resilience Scale; MBI, Maslach Burnout Inventory, EE—emotional exhaustion, PA—personal accomplishment; MITS, Mindfulness in Teaching Scale; PSS, Perceived Stress Scale; ** Correlation is significant at the 0.01 level. * Correlation is significant at the 0.05 level.

## Data Availability

The data presented in this study are available on request from the corresponding author. The data are not publicly available due to Israeli Ministry of Education confidential policies.

## Supplementary Materials

The following are available online at https://www.mdpi.com/article/10.3390/ijerph18073689/s1, Table S1: Judge Your Neighbor Worksheet; Table S2: Differences between study participants and dropouts; Table S3: Cronbach’s Alpha of research questionnaires; Table S4: Differences in baseline outcomes between younger (age ≤ 46 years) and elder (age > 46 years) participants; Table S5: Differences in baseline outcomes between male and female participants.
