# Supplementary material for: Effect of Inquiry-Based Stress Reduction (IBSR) Intervention on Well-Being, Resilience and Burnout of Teachers during the COVID-19 Pandemic
Source: Int J Environ Res Public Health. 2021 Apr 1;18(7):3689. doi: 10.3390/ijerph18073689 (PMC8037267; doi:10.3390/ijerph18073689)
Supplement: Supplementary file 1 [file ijerph-18-03689-s001.pdf]

Supplementary Materials: Table S1
Judge Your Neighbor Worksheet

Judge-Your-Neighbor Worksheet

Think of a stressful situation with someone—for example, an argument. As you meditate on that specific time and place and begin to feel what that felt like, fill in the blanks below. Use short, simple sentences.

1. **In this situation, who angers, confuses, hurts, saddens, or disappoints you, and why?**

   I am ____________ with ______________ because ______________________________
   emotion / name
   ____
   I am angry with Paul because he lied to me.

WANTS 2. **In this situation, how do you want him/her to change? What do you want him/her to do?**

   I want ______________ to ______________________________
   name
   ____
   ____
   I want Paul to see that he is wrong. I want him to stop lying to me.

ADVICE 3. **In this situation, what advice would you offer him/her? "He/she should/shouldn't..."**

   ______________ should/shouldn't ______________________________
   name
   ____
   ____
   ____
   Paul shouldn't frighten me with his behavior. He should take a deep breath.

NEEDS 4. **In order for *you* to be happy in this situation, what do you need him/her to think, say, feel, or do?**

   I need ______________ to ______________________________
   name
   ____
   ____
   I need Paul to stop talking over me. I need him to really listen to me.

COMPLAINTS 5. **What do you think of him/her in this situation? Make a list.** (It's okay to be petty and judgmental.)

   ______________ is ______________________________
   name
   ____
   Paul is a liar, arrogant, loud, dishonest, and unconscious.

6. **What is it about this person and situation that you don't ever want to experience again?**

   I don't ever want ______________________________
   ____
   I don't ever want Paul to lie to me again. I don't ever want to be disrespected again.

Now question each of your statements, using the four questions of The Work, below. For the turnaround to statement 6, replace the words *I don't ever want...* with *I am willing to...* and *I look forward to...*

The four questions

Example: Paul lied to me.

1. Is it true? (Yes or no. If no, move to question 3.)
2. Can you absolutely know that it's true? (Yes or no.)
3. How do you react, what happens, when you believe that thought?
4. Who or what would you be without the thought?

Turn the thought around.

I lied to me.
I lied to *Paul.*
Paul *didn't* lie to me.
Paul *told me the truth.*

As you visualize the situation, contemplate how each turnaround is as true or truer.

THE WORK OF BYRON KATIE © 2019 Byron Katie International, Inc. All rights reserved. thework.com

6 Feb. 2019

Table S2.
Differences between study participants and dropouts.

		Study participants (N= 60)	Dropouts (N=7)	T-test between groups	
		**Mean (SD)**	**Mean (SD)**	**Test statistic**	**P value**
**Age (yrs)**		45.3 (9.1)	42.4 (10.6)	T= 0.79	0.431
**Education (yrs)**		17.5 (2.6)	18.9 (7.4)	T= 1.04	0.303
**Seniority[a] (yrs)**		16.3 (9.7)	15.4 (10.7)	T= 0.21	0.832
**Job percent (%)**		92.5 (14.3)	92.3 (7.6)	T= 0.03	0.973
**Gender[b]**	Female	52 (86.7%)	6 (85.7%)	X^2= 0.00[a]	0.944
	Male	8 (13.3%)	1 (14.3%)		
**Marital status[b]**	Single	5 (8.3%)	2 (28.6%)	X^2= 5.09[a]	0.278
	Married without children	3 (5.0%)	0 (0.0%)		
	Married with children	44 (73.3%)	3 (42.9%)		
	Divorced	7 (11.7%)	2 (28.6%)		
	widowed	1 (1.7%)	0 (0.0%)		
**Economic[b]**	Below average	5 (30%)	1 (14.3%)		0.641
	Average	37 (61.7%)	5 (71.4%)		
	Above average	18 (8.3%)	1 (14.3%)		
**PERMA**		156.2 (20.2)	160.4 (18.6)	T= 0.52	0.604
**SWLS**		25.5 (5.4)	26.0 (4.8)	T= 0.23	0.821
**PANAS-P**		39.4 (5.4)	36.7 (5.7)	T= 1.22	0.227
**PANAS-N**		18.5 (6.1)	18.4 (4.9)	T= 0.04	0.963
**BRS**		3.3 (0.8)	3.4 (0.5)	T= 0.17	0.865
**MBI EE**		11.3 (5.6)	16.3 (6.2)	T= 2.19	0.032*
**MBI PA**		9.2 (5.3)	12.4 (6.9)	T= 1.48	0.143
**MITS**		74.5 (7.2)	77.4 (5.7)	T= 1.04	0.300
**PSS**		36.5 (10.2)	31.4 (10.7)	T= 1.24	0.216

Abbreviations: PERMA, positive emotion, engagement, relationships, meaning, and accomplishment; SWLS, Satisfaction with Life Scale; PANAS, Positive and Negative Affect Scale; BRS, Brief Resilience Scale; MBI, Maslach Burnout Inventory, EE- emotional exhaustion, PA- personal accomplishment; MITS, Mindfulness in Teaching Scale; PSS, Perceived Stress Scale;
ANOVA, analysis of variance; SD, standard deviation.
*** p-value of <0.05 indicating statistical significance.**
[a] How many years have you been in the teaching profession?
[b] N (% within group)

Table S3

Differences in baseline outcomes between younger (age≤46 years) and elder (age>46 years) participants.

	Younger participants (N=34)	Elder Participants (N=33)	Difference between groups	
	**Mean (SD)**	**Mean (SD)**	**Test statistic**	**P value**
**PERMA**[d]	153.9 (21.9)	159.7 (17.5)	F= 1.39	0.244
**SWLS**[b,d]	26.4 (5.6)	24.8 (4.9)	F= 1.53	0.220
**PANAS-P**[a,c]	57.7 (7.6)	61.9 (6.9)	F= 4.70	0.034*
**PANAS-N**[d]	17.6 (5.9)	19.5 (5.9)	F= 1.65	0.204
**BRS**[d]	3.4 (0.8)	3.3 (0.9)	F= 0.46	0.502
**MBI EE**	12.4 (5.9)	11.3 (5.7)	F= 0.60	0.441
**MBI PA**	9.7 (5.5)	9.3 (5.6)	F= 0.08	0.785
**MITS**	74.9 (7.7)	74.6 (6.6)	F= 0.04	0.849
**PSS**	35.6 (10.7)	36.4 (9.9)	F= 0.08	0.778

Abbreviations: PERMA, positive emotion, engagement, relationships, meaning, and accomplishment; SWLS, Satisfaction with Life Scale; PANAS, Positive and Negative Affect Scale; BRS, Brief Resilience Scale; MBI, Maslach Burnout Inventory, EE- emotional exhaustion, PA- personal accomplishment; MITS, Mindfulness in Teaching Scale; PSS, Perceived Stress Scale;

*** p-value of <0.05 indicating statistical significance.**

N (% within group)

[a] N (Younger)= 30

[b] N (Younger)= 33

[c] N (Elder)= 30

[d] N (Elder)= 32

Table S4

Differences in baseline outcomes between male and female participants.

	Female participants (N=58)	Male Participants (N=9)	t-test between groups	
	Mean (SD)	Mean (SD)	Test statistic	P value
**PERMA**[a]	156.4 (20.2)	158.4 (19.1)	T= 0.25	0.800
**SWLS**[a.b]	25.2 (5.3)	28.4 (4.1)	T= 1.63	0.109
**PANAS-P**[b]	39.0 (5.6)	39.3 (4.7)	T= 0.15	0.880
**PANAS-N**[b]	18.9 (6.2)	15.8 (2.8)	T= -1.49	0.139
**BRS**[a]	3.3 (0.8)	3.7 (0.5)	T= 1.48	0.143
**MBI EE**	12.2 (5.8)	9.5 (5.6)	T= -1.26	0.210
**MBI PA**	9.8 (5.6)	7.8 (4.6)	T= -1.02	0.313
**MITS**	74.9 (7.1)	73.8 (7.4)	T= -0.45	0.654
**PSS**	35.4 (10.4)	39.5 (8.9)	T= 1.12	0.268

Abbreviations: PERMA, positive emotion, engagement, relationships, meaning, and accomplishment; SWLS, Satisfaction with Life Scale; PANAS, Positive and Negative Affect Scale; BRS, Brief Resilience Scale; MBI, Maslach Burnout Inventory, EE- emotional exhaustion, PA- personal accomplishment; MITS, Mindfulness in Teaching Scale; PSS, Perceived Stress Scale;

*** p-value of <0.05 indicating statistical significance.**

N (% within group)

[a] N (Male)= 8

[b] N (Female)= 57

Table S5
Cronbach's Alpha of research questionnaires.

	Cronbach's Alpha before
**PERMA**	α = 0.812
**SWLS**	α = 0.810
**PANAS-P**	α = 0.770
**PANAS-N**	α = 0.841
**BRS**	α = 0.824
**MBI EE**	α = 0.877
**MBI PA**	α = 0.799
**MITS**	α = 0.707
**PSS**	α = 0.859

Abbreviations: PERMA, positive emotion, engagement, relationships, meaning, and accomplishment; SWLS, Satisfaction with Life Scale; PANAS, Positive and Negative Affect Scale; BRS, Brief Resilience Scale; MBI, Maslach Burnout Inventory, EE- emotional exhaustion, PA- personal accomplishment; MITS, Mindfulness in Teaching Scale; PSS, Perceived Stress Scale;
